# A Novel C-Terminal Domain of RecJ is Critical for Interaction with HerA in *Deinococcus radiodurans*

**DOI:** 10.3389/fmicb.2015.01302

**Published:** 2015-11-30

**Authors:** Kaiying Cheng, Ye Zhao, Xuanyi Chen, Tao Li, Liangyan Wang, Hong Xu, Bing Tian, Yuejin Hua

**Affiliations:** Key Laboratory of Chinese Ministry of Agriculture for Nuclear-Agricultural Sciences, Institute of Nuclear-Agricultural Sciences, Zhejiang UniversityHangzhou, China

**Keywords:** RecJ, *Deinococcus radiodurans*, HerA, NurA, DNA end resection, protein–protein interaction, DNA repair

## Abstract

Homologous recombination (HR) generates error-free repair products, which plays an important role in double strand break repair and replication fork rescue processes. DNA end resection, the critical step in HR, is usually performed by a series of nuclease/helicase. RecJ was identified as a 5′-3′ exonuclease involved in bacterial DNA end resection. Typical RecJ possesses a conserved DHH domain, a DHHA1 domain, and an oligonucleotide/oligosaccharide-binding (OB) fold. However, RecJs from *Deinococcus-Thermus* phylum, such as *Deinococcus radiodurans* RecJ (DrRecJ), possess an extra C-terminal domain (CTD), of which the function has not been characterized. Here, we showed that a CTD-deletion of DrRecJ (DrRecJΔC) could not restore *drrecJ* mutant growth and mitomycin C (MMC)-sensitive phenotypes, indicating that this domain is essential for DrRecJ *in vivo*. DrRecJΔC displayed reduced DNA nuclease activity and DNA binding ability. Direct interaction was identified between DrRecJ-CTD and DrHerA, which stimulates DrRecJ nuclease activity by enhancing its DNA binding affinity. Moreover, DrNurA nuclease, another partner of DrHerA, inhibited the stimulation of DrHerA on DrRecJ nuclease activity by interaction with DrHerA. Opposing growth and MMC-resistance phenotypes between the *recJ* and *nurA* mutants were observed. A novel modulation mechanism among DrRecJ, DrHerA, and DrNurA was also suggested.

## Introduction

RecJ is a Mg^2+^/Mn^2+^ dependent 5′-3′ single-stranded DNA (ssDNA) specific exonuclease in the RecJ/DHH protein superfamily (Aravind and Koonin, 1998). RecJ/DHH family members contain conserved DHH domain, which was named after three characteristic amino acids (Aravind and Koonin, 1998). Appearing in almost all prokaryotes and eukaryotes implies a fundamental role of these family members (Aravind and Koonin, 1998; Rajman and Lovett, 2000; Sanchez-Pulido and Ponting, 2011; Krastanova et al., 2012; Makarova et al., 2012; Sarmiento et al., 2014). The typical RecJ proteins exist in almost all eubacteria. They possess a DHH, a DHHA1 and an oligonucleotide/oligosaccharide-binding (OB) fold domain, among which the DHH and DHHA1 domains form the catalytic core, and the OB fold assists in DNA binding (Aravind and Koonin, 1998; Wakamatsu et al., 2010). Moreover, the RecJs in *Deinococcus-Thermus* phylum exhibit an additional uncharacterized C-terminal domain (CTD) (Wakamatsu et al., 2011; Jiao et al., 2012). RecJ is believed to be involved in a number of processes *in vivo*, including resects DNA end in the RecFOR homologous recombination (HR) pathway (Handa et al., 2009a,b; Morimatsu and Kowalczykowski, 2014), mediates the excision step during mismatch repair (Burdett et al., 2001; Viswanathan et al., 2001), degrades abasic residues during base excision repair (Dianov et al., 1994), reduces homology-facilitated illegitimate recombination events (Ukita and Ikeda, 1996; Harms et al., 2007), and rescues stalled replication forks (Courcelle and Hanawalt, 1999, 2001; Courcelle et al., 2003; Rudolph et al., 2008). Numerous studies suggest that RecJ and RecBCD might have overlapping functions. In *Escherichia coli* and *Salmonella typhimurium, recB* and *recJ* double mutations resulted in recombination deficiency (Lovett and Clark, 1984; Garzon et al., 1996). Both *recBCD* mutant and *recJ* mutant have moderate phenotypes in *Acinetobacter baylyi*, while the double mutant is lethal (Kickstein et al., 2007). *Deinococcus radiodurans*, an extremely radioresistant bacterium naturally lacking RecBCD (Cox et al., 2010), showed remarkable growth defects and sensitive to high temperatures when its *recJ* was disrupted (Jiao et al., 2012). RecJ was reported to be co-purified by single-stranded DNA binding protein (SSB) in *E. coli* (Butland et al., 2005) and its core domain was confirmed to directly interact with the C-termini of SSB in *Haemophilus influenza* (Sharma and Rao, 2009). The ssDNA nuclease activity of RecJ could be stimulated by SSB through enhancing DNA binding efficiency (Han et al., 2006; Sharma and Rao, 2009). We previous showed that in *D. radiodurans*, the RecJ nuclease activity could be enhanced by SSB as well (Jiao et al., 2012).

The orthologues of HerA proteins are highly conserved among archaea, which also present in some bacteria but absent in eukaryotes (Iyer et al., 2004). However, the biological functions of HerA have not been well characterized. HerA belongs to the FtsK-HerA superfamily of P-loop ATPases (Iyer et al., 2004). The structural and evolutionary relationship between HerA and FtsK and the nearly perfect complementarity of their phyletic distributions suggest that HerA might have similar functions as FtsK, including mediating DNA pumping into the progeny cells during cell division (Iyer et al., 2004). In archaea, HerA was reported to be an ATPase with bidirectional helicase activity (Constantinesco et al., 2004; Manzan et al., 2004). The *nurA* gene, which encodes a 5′-3′ ss/dsDNA exonuclease/endonuclease NurA, is usually located in the same operon with *herA* gene (Constantinesco et al., 2002). Structural and functional relationships between HerA and NurA have been confirmed recently (Hopkins and Paull, 2008; Blackwood et al., 2012; Byrne et al., 2014; Rzechorzek et al., 2014). In Archaea, HerA and NurA are located in the same operon with Mre11 and Rad50, suggesting these four proteins might work together in DNA repair (Hopkins and Paull, 2008; Quaiser et al., 2008). Recently, we characterized HerA and NurA from *D. radiodurans* (Cheng et al., 2015). The HerA and NurA showed similar biochemical activities as archaeal HerA and NurA. Decreased intermolecular recombination efficiency was confirmed in the HerA and NurA mutant strains. However, little contribution of HerA and NurA to the radioresistance of *D. radiodurans* was observed (Cheng et al., 2015).

In this study, we demonstrated that *D. radiodurans* RecJ (DrRecJ) lacking CTD could not fully restore the *drrecJ* knockout strain. A direct interaction between DrRecJ-CTD and DrHerA was confirmed by far western blotting assays and pull-down assays. The functional relationship between DrHerA and DrRecJ was further analyzed. Moreover, DrNurA, another interaction partner of DrHerA, showed strong inhibitory effect against DrHerA stimulation of DrRecJ.

## Results

### DrRecJΔC could not Compensate for the Cell Growth and MMC Resistance Defect of *recJ* Mutant

**FIGURE 1:**
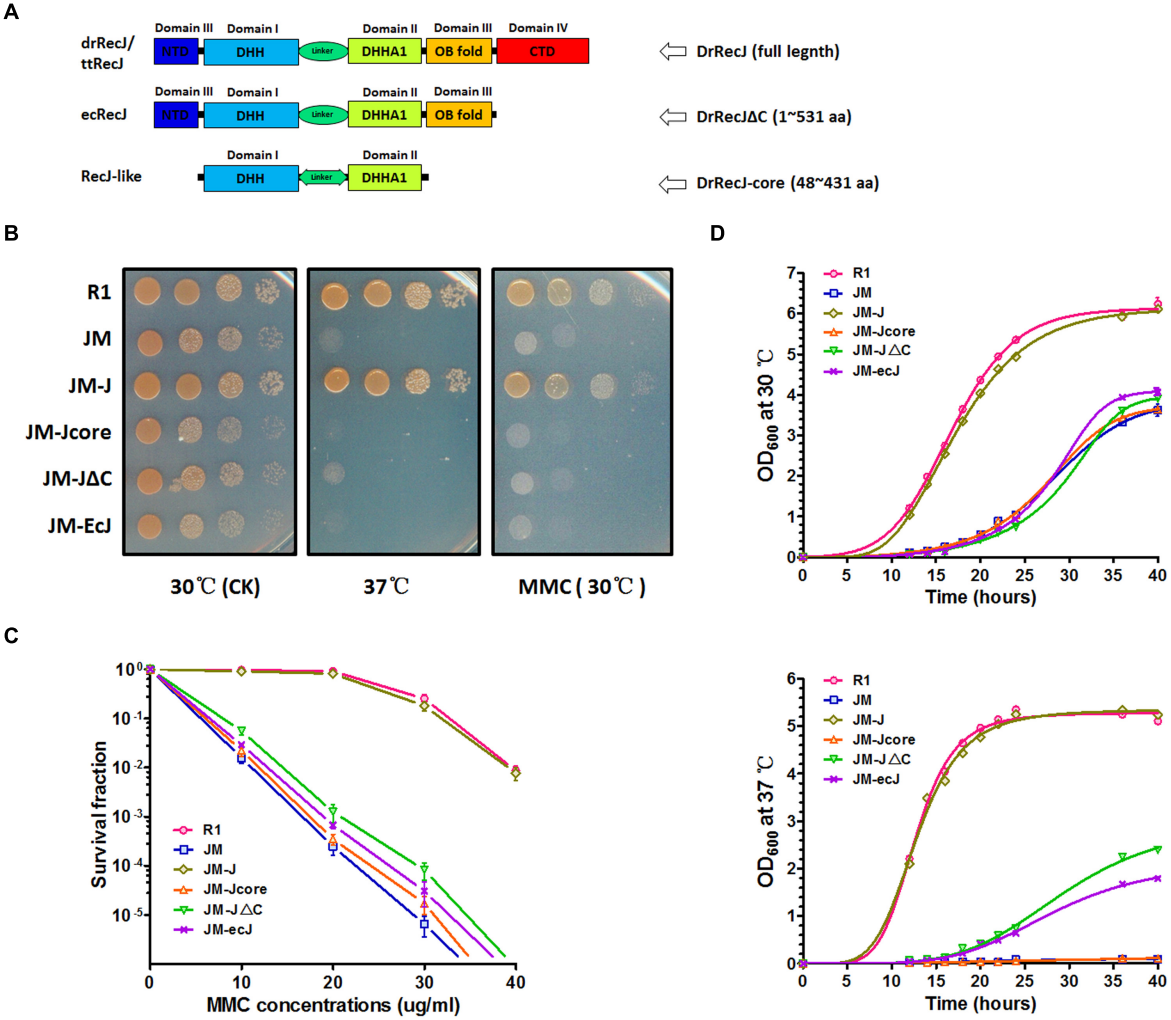
**Phenotypes of *recJ* knockout strain and complemented strains. (A)** Schematic representations of RecJ/DHH protein superfamily. Each domain is colored ranging from blue at the N terminus to red at the C terminus based on the structure of ttRecJ (PDB code: 2ZXP). Truncations of DrRecJ were constructed according to the domains of EcRecJ and RecJ-like protein. **(B)** Growth and MMC resistance features of different strains. The diluted bacteria suspensions (treated with 20 ug ml^-1^ MMC or not) of wild type strains (R1), *drrecJ* disruptant (JM), JM with *drrecJ* complemented (JM-J), JM with *drrecJΔC* complemented (JM-JΔC), JM with *drrecJ-core* complemented (JM-Jcore), and JM with *ecrecJ* complemented (JM-ecJ) were grown to the same OD_600_, spotted on TGY medium and cultured at 30°C and 37°C. **(C)** The survival fractions of different strains with different MMC doses treatments. The survival fraction curves were plotted using GraphPad Prism 5 software. The data of R1, JM, JM-J, and JM-JΔC were marked with light red circle, light green square, light blue triangle and light hollow inverted triangle, respectively. Data show mean values from three independent experiments and bars depict the standard deviation (SD). **(D)** Growth curves of different strains at different temperatures. The OD_600_ value of each strain was measured at different time points. The growth curves were plotted using GraphPad Prism 5 software. Data shown mean values from three independent experiments and bars depict the standard deviation (SD). Up: assay tested at 30°C; down: assay tested at 37°C.

RecJ is a DNA exonuclease belonging to the RecJ/DHH superfamily of phosphoesterases. Members of this superfamily usually possess a conserved DHH domain (Domain I, motifs A–D) and a DHHA1 domain (Domain II, motifs E and F) (Supplemental Figure S1A). Besides these two representative domains, typical RecJ proteins always possess a conserved OB fold (domain III). Moreover, RecJs from *Deinococcus-Thermus* phylum have extra conserved CTDs (domain IV) with more than 20% sequential identities (**Figure 1A**; Supplemental Figure S1B). However, these CTDs are uncharacterized, which have no defined homolog as analyzed by the HHpred online tool.

In order to investigate the function of RecJ-CTD in *D. radiodurans*, we constructed a CTD-deleted DrRecJ (DrRecJΔC) complementation strain and an OB fold/CTD-deleted DrRecJ (DrRecJ-core) complementation strain. Truncations of DrRecJ were constructed according to the sequence alignments with EcRecJ and RecJ-like proteins (**Figure 1A**), and detailed information was shown in “Materials and Methods.” In addition, an EcRecJ complementation strain was also constructed to test if EcRecJ could substitute for DrRecJ *in vivo*. Western blotting results indicated that the complemented DrRecJΔC, DrRecJ-core, and EcRecJ were expressed *in vivo* (Figure S1C). Cell growth and cell survival rates with MMC treatment were also compared. The *drrecJ* mutant grew much more slowly than the wild type strain, especially at high growth temperature (37°C) (**Figures 1B,D**). The *drrecJ* mutant was highly sensitive to MMC treatment (**Figure 1B**). After treatment with MMC (20 μg mL^-1^) for 20 min, the survival rate of the dr*recJ* mutant was up to thousand fold lower than the wild type strain (**Figure 1C**). The addition of full length DrRecJ could completely restore the growth and MMC-resistance defects, while DrRecJΔC, DrRecJ-core or EcRecJ could not (**Figures 1B–D**), indicating that the CTD is critical for DrRecJ function *in vivo*.

### DrRecJΔC Displays Reduced Nuclease Activity and DNA Binding Ability

**FIGURE 2:**
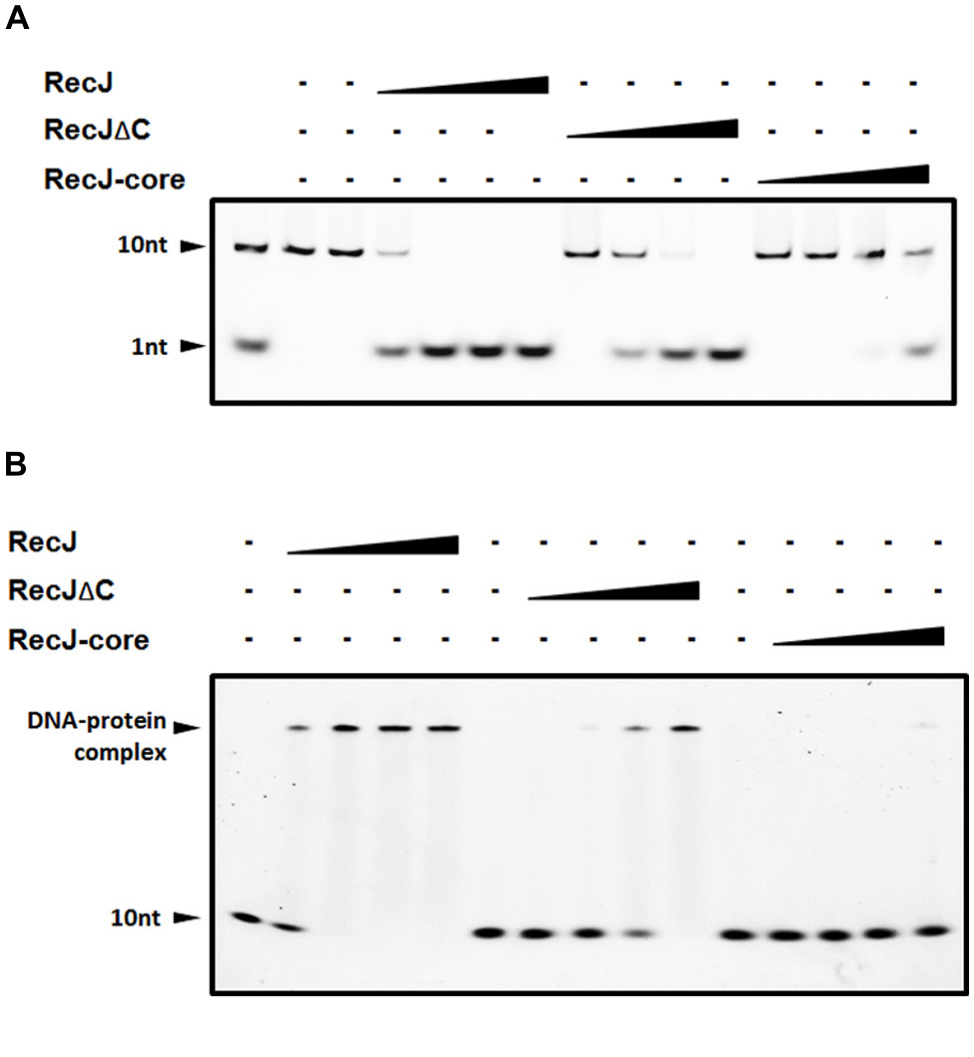
**Comparisons of nuclease activity and ssDNA binding ability among DrRecJ, DrRecJΔC and DrRecJ-core. (A)** Nuclease activities of DrRecJ, DrRecJΔC and DrRecJ-core. Hundred nanomolar 10 nt ssDNA was used as substrate for RecJ digestion. The molar ratio of ssDNA:DrRecJ (or DrRecJΔC, DrRecJ) = 5:1, 5:4, 5:16, 5:64. **(B)** ssDNA binding ability of DrRecJ, DrRecJΔC and DrRecJ-core. Hundred nanomolar 10 nt ssDNA was used as substrate for RecJ binding. The molar ratio of ssDNA:RecJ (or DrRecJΔC, DrRecJ) = 1:2, 1:4, 1:8, 1:16.

The OB fold of RecJ was confirmed to assist DNA binding in *T. thermophilus* (Wakamatsu et al., 2010). However, the function of CTD has not been characterized. We purified DrRecJ, DrRecJΔC, and DrRecJ-core (Supplemental Figure S2) and tested their nuclease activities and DNA binding activities respectively. Compared with DrRecJ, both DrRecJ-core and DrRecJΔC showed reduced nuclease activity (**Figure 2A**). Similarly, both DrRecJ-core and DrRecJΔC showed much weaker ssDNA affinity than full length DrRecJ, which was in agreement with those of the nuclease activity (**Figure 2B**). These results indicate that the CTD of DrRecJ also contributes to RecJ DNA binding capability.

### DrRecJ Interact with DrHerA through the CTD

**FIGURE 3:**
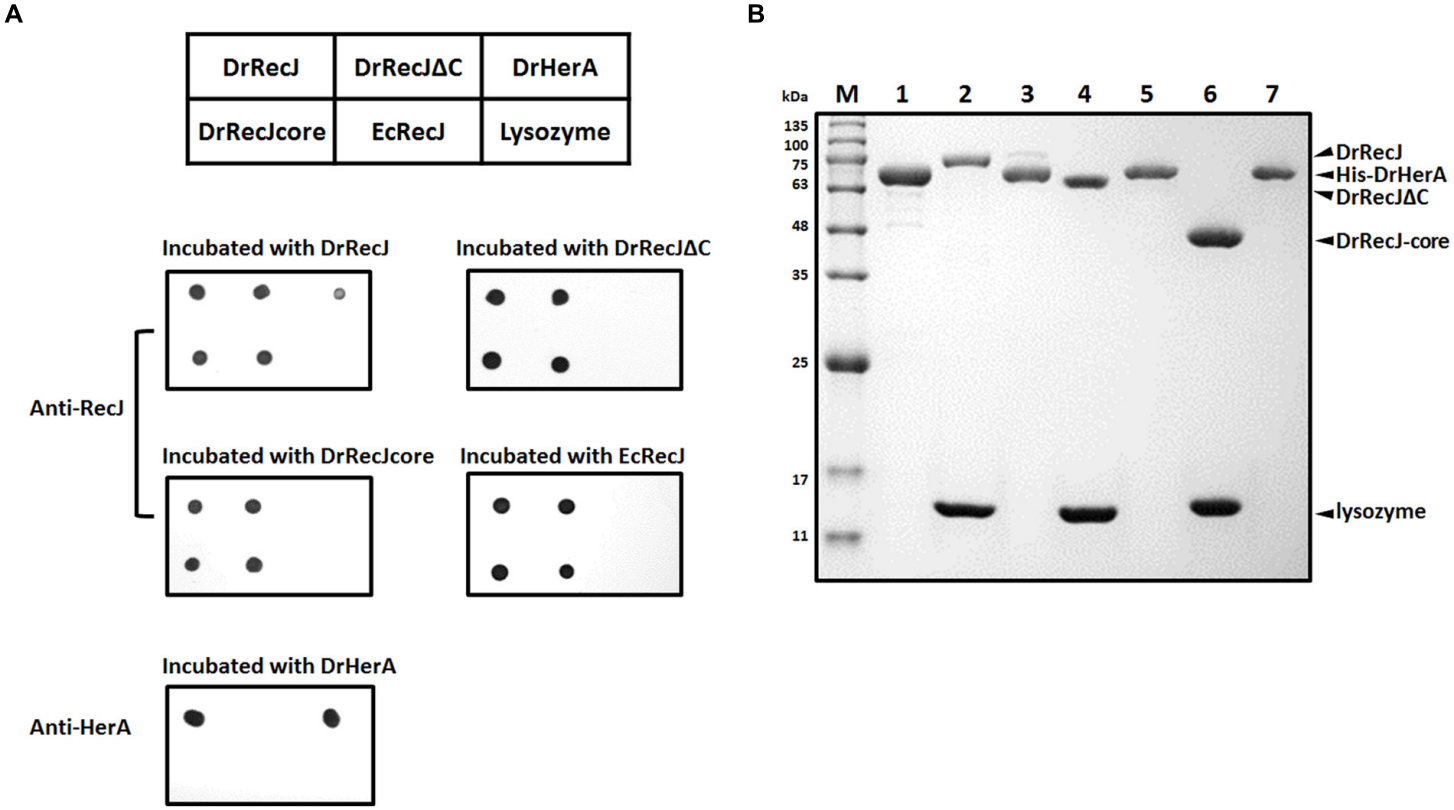
**Interaction assays between DrRecJ and DrHerA. (A)** Far western blotting assays. The table above represents the orders of different proteins dotted on the NC membranes. Each membrane was incubated with different proteins and checked by anti-RecJ or anti-HerA antibody. **(B)** Pull down assays. Four micro liter 0.5 mM RecJ, RecJΔC or RecJ-core (with lysozyme as control) was incubated with His-DrHerA bound Ni-NTA beads and washed until input control be washed off, and analyzed by 12% SDS-PAGE. Lane 1: His-DrHerA; Lane 2: DrRecJ + lysozyme (input control); Lane 3: DrRecJ pulled by His-DrHerA; Lane 4: DrRecJΔC + lysozyme (input control); Lane 5: DrRecJΔC pulled by His-DrHerA; Lane 6: DrRecJ-core + lysozyme (input control); Lane 7: DrRecJ-core pulled by His-DrHerA.

That DrRecJΔC could not fully compensate for the mutant defect also promotes us to find out whether the DrRecJ-CTD participates in the interactions with other important proteins *in vivo*. Total protein extracted from *D. radiodurans* was incubated with anti-DrRecJ antibody bound protein G beads. The coimmunoprecipitated proteins were concentrated and analyzed by SDS-PAGE (Supplemental Figure S3), followed by identification using mass spectrometry. In addition to DrSSB, DrHerA was also identified as a potential interaction partner of DrRecJ. A direct interaction between DrRecJ and DrHerA was confirmed by far western blotting assays. No interactions between DrHerA-DrRecJΔC, DrHerA-DrRecJ-core, or DrHerA-EcRecJ were observed (**Figure 3A**), suggesting that the CTD of DrRecJ was the major interaction site. Pull-down assays were also carried out. HerA protein with N-terminal His-tag (His-HerA) was incubated with Ni-NTA beads. The full length DrRecJ rather than truncated DrRecJ (DrRecJΔC or DrRecJ-core) was pulled down by His-HerA (**Figure 3B**), indicating that DrRecJ and DrHerA interact with each other through the CTD of DrRecJ.

### DrHerA Enhances DrRecJ Nuclease Activity and ssDNA Binding Ability

**FIGURE 4:**
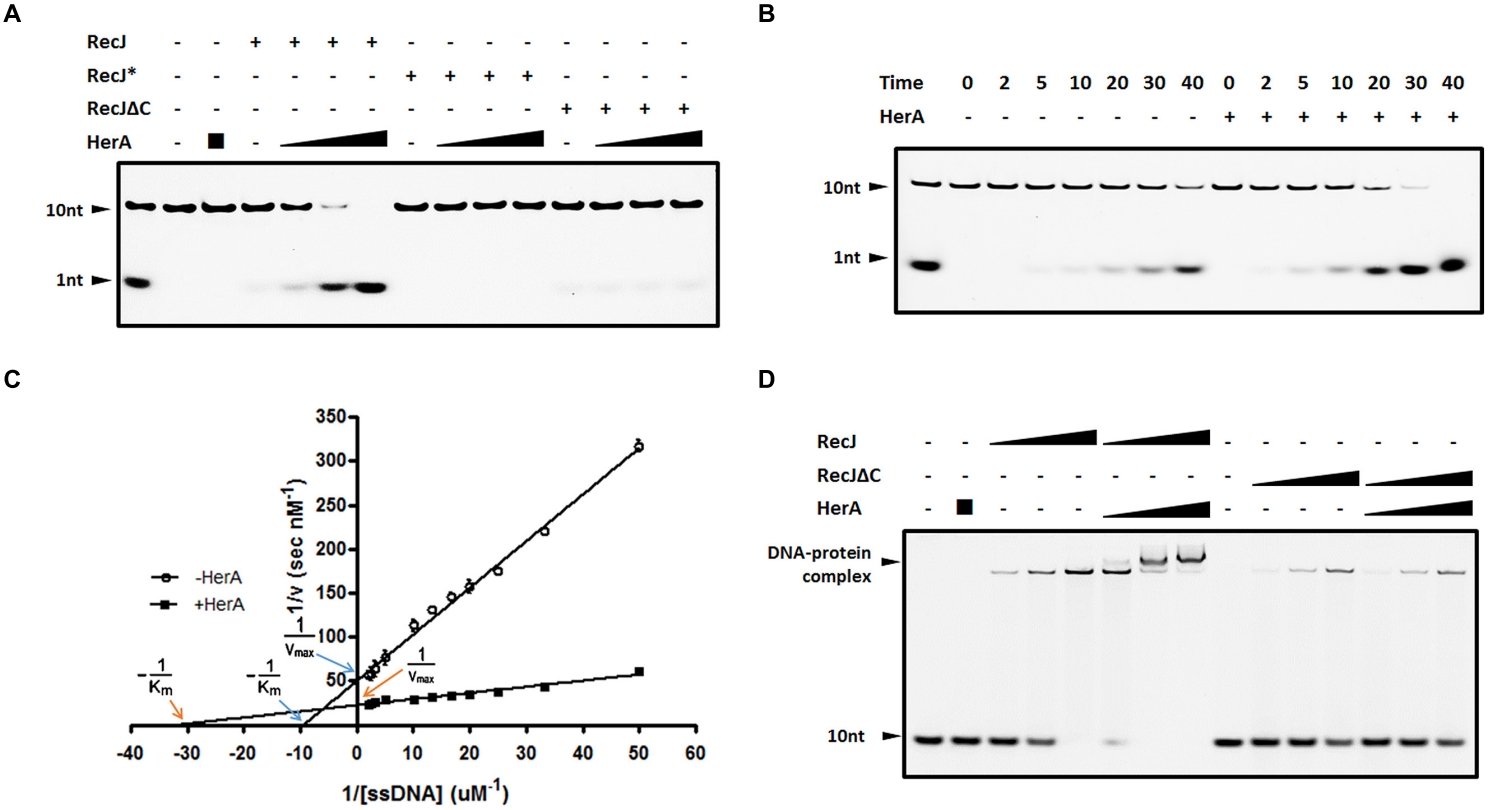
**DrHerA enhanced DrRecJ nuclease activity and ssDNA binding ability. (A)** DrHerA enhanced DrRecJ nuclease activity. Hundred nanomolar 10 nt ssDNA was used as substrate for RecJ digestion. DrRecJ ssDNA nuclease activity was analyzed in the absence or presence of DrHerA in various molar ratios (RecJ monomer: HerA hexamer = 1:1, 1:4, 1:16). RecJ^∗^ represented the inactive DrRecJ protein DrRecJ (D158A/H159A/H160A). Five nanomolar DrRecJ was used while 40 nM DrRecJΔC was used in the reaction system. **(B)** Time course experiments for DrHerA enhancement on DrRecJ nuclease activity. DNA hydrolysis by DrRecJ in the presence or absence of DrHerA was analyzed at different time points (2, 5, 10, 20, 30, and 40 min). **(C)** Steady-state kinetics analyses of DNA hydrolysis by DrRecJ in the presence or absence of DrHerA. The amount of undegraded substrate remaining for each concentration was quantitated and used to calculate the velocity (v) of the reaction, the reciprocal of which was plotted against the reciprocal of substrate concentration (1/[v] versus 1/[S]). Lineweaver–Burk equation was used for the calculation of kinetic parameters. Error bars indicate standard deviation. **(D)** DrHerA enhanced DrRecJ ssDNA binding activity. Hundred nanomolar 10 nt ssDNA was used as substrate for RecJ binding. The molar ratio: DNA: DrRecJ = 1:1, 1:2, 1:4; DNA: DrRecJΔC = 1:4, 1:8, 1:16; HerA (hexamer): DrRecJ (or DrRecJΔC) = 8: 1.

Since DrHerA could directly interact with DrRecJ, we tested the nuclease activity of DrRecJ in the presence and absence of DrHerA. DrHerA was pre-incubated with DrRecJ to allow the formation of DrRecJ–DrHerA complexes prior to the addition of 10 nt 5′ FAM-labeled ssDNA. The reaction was initiated by the addition of 0.1 mM Mn^2+^. Along with adding increasing amounts of DrHerA, increasing amounts of processed substrate (1 nt band) was observed, indicating that DrRecJ activity could be stimulated by DrHerA *in vitro* (**Figure 4A**). Such stimulations were further confirmed by time course experiments (**Figure 4B**). No obvious stimulation of DrRecJΔC was observed (**Figure 4A**), indicating that such stimulation came from direct interactions between DrRecJ-CTD and DrHerA. Stimulations by DrHerA were also observed using other DNA substrates such as longer ssDNA (46 nt) and 5′ overhanging DNA (Supplemental Figure S4A). Furthermore, DrHerA showed no stimulation of the *D. radiodurans* DrRecJ-like protein (Dr_0826) or EcRecJ activity (Supplemental Figure S4B), implying this stimulation is protein-specific and species-specific.

The steady state kinetics of ssDNA degradation by DrRecJ, with or without DrHerA, was measured. Different concentrations of 10 nt 5′ FAM-labeled ssDNA (20∼500 nM) were incubated with 5 nM RecJ, in the presence or in the absence of 500 nM DrHerA. The reaction rate [v] was estimated from quantitation of the amount of undegraded substrate remaining for each substrate concentration. The plot of 1/[v] versus 1/[S] showed well fit to the Lineweaver–Burk equation and was used to estimate *K_m_* and *V_max_* values (**Figure 4C**). The *K_m_* for DrRecJ was estimated to be 0.1 μM in the absence of DrHerA and 0.031 μM in the presence of DrHerA. The *V_max_* for DrRecJ was estimated to be 0.2 (nM S^-1^) in the absence of DrHerA and 0.4 (nM S^-1^) in the presence of DrHerA. When DrHerA presented, the decreased *K_m_* value and increased *V_max_* value indicates that the affinity of RecJ for substrate DNA was enhanced in the presence of DrHerA. The DNA binding abilities of DrRecJ in the absence and presence of DrHerA were also compared. While DrHerA alone could not bind 10 nt ssDNA, a super-shift of HerA-RecJ-DNA complex was observed, indicating that DrHerA could enhance DrRecJ ssDNA binding ability (**Figure 4D**). For DrRecJΔC, no obvious enhancement was observed, indicating such stimulation was mediated by DrRecJ-CTD (**Figure 4D**).

### DrNurA Blocks DrHerA Stimulation of DrRecJ Nuclease Activity by Interacting with DrHerA

**FIGURE 5:**
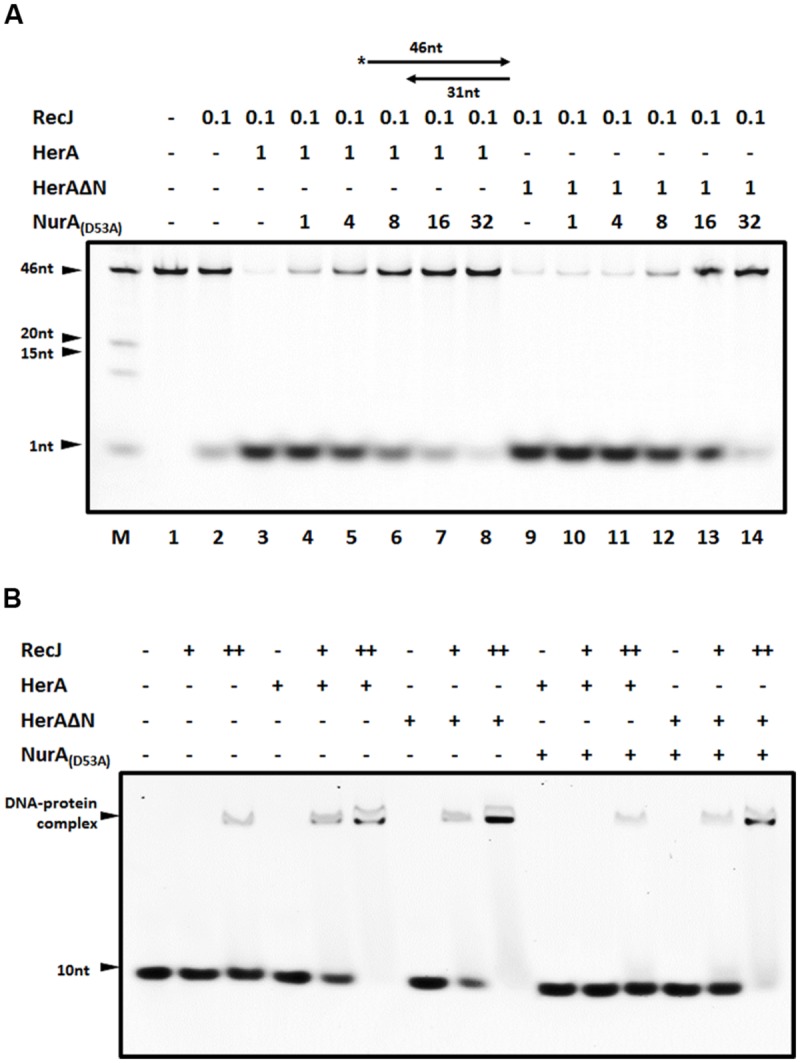
**The blockage of DrNurA on DrHerA stimulation on DrRecJ nuclease activity and DNA binding ability. (A)** DrNurA inhibited DrHerA stimulation on DrRecJ nuclease activity. Hundred nanomolar 5′ overhang DNA (annealed by O2 and O3) was used as substrate and digested by 5 nM DrRecJ. Various concentrations of DrNurA were added in the reaction system (HerA hexamer: NurA dimer = 1:1, 1:4, 1:8, 1:16, 1:32). DrRecJ ssDNA nuclease activity was analyzed in the absence or presence of DrHerA (DrHerA ΔN) in molar ratios (RecJ monomer: HerA hexamer = 1:8). **(B)** NurA inhibit HerA stimulation on RecJ ssDNA binding activity while DrHerA ΔN do not. Hundred nanomolar 10 nt ssDNA was used as substrate for DrRecJ binding. Fivety nanomolar or 100 nM RecJ was used in the binding assays. Eight hundred nanmolar DrHerA (hexamer) or DrHerA ΔN (hexamer), or 4 μM DrNurA (dimer) was added, if necessary.

DrNurA is another DrHerA interaction partner with nuclease activity, which can be stimulated by DrHerA (Cheng et al., 2015). Given that both DrNurA and DrRecJ possess 5′-3′ ssDNA exonuclease activity and have physical/functional relationships with DrHerA, we were particularly interested in the possible interplay of these three proteins. In order to mimic the 3′ end resection process *in vivo*, a 5′ overhanging DNA substrate was used. The digestion efficiency of DrRecJ was highly elevated in the presence of DrHerA (**Figure 5A**, lane 3). However, the addition of catalytic inactive DrNurA mutant (D53A) impaired the stimulation (**Figure 5A**, lanes 4–8). DrHerAΔN, which still forms a hexametric ATPase but no longer interacts with NurA, could also stimulate the nuclease activity of DrRecJ (**Figure 5A**, lane 9). The addition of equal amount of DrNurA (D53A) could not inhibit the stimulation (**Figure 5A**, lanes 10–14). These results suggest DrNurA could competitively bind to DrHerA, thus reducing the stimulation of DrRecJ nuclease activity. However, the DrRecJ and DrNurA appear to interact with DrHerA with different sites because DrHerAΔN could still interact with DrRecJ (Supplemental Figure S5A). Moreover, pull down assays were carried out among DrRecJ, DrHerA, and DrNurA, showing co-binding band of these three proteins (Supplemental Figure S5B, lane 7). Therefore, DrNurA compete with DrRecJ for DrHerA binding is not likely. The addition of DrNurA decreased DrHerA but not DrHerAΔN stimulation of DrRecJ on DNA binding (**Figure 5B**), indicating that the co-binding of DrNurA on RecJ-HerA reduced the substrate affinity or digestion of RecJ. On the other hand, overwhelming DrNurA strongly inhibits the intrinsic nuclease activity of DrRecJ even if DrHerA or DrHerAΔN were present (**Figure 5A**, lanes 8 and 14). Reactions without DrHerA were also carried out and showed that high concentrations of DrNurA could inhibit DrRecJ nuclease activity (Supplemental Figure S5C). Moreover, catalytic inactive DrRecJ also inhibits DrHerA stimulation on DrNurA nuclease activity (Supplemental Figure S5D). These results suggest that, in addition to reduce DrHerA stimulation of DrRecJ activity, DrNurA also has weak substrate competition activity with RecJ.

### The recJ Mutant and nurA Mutant Display Opposite Phenotypes

**FIGURE 6:**
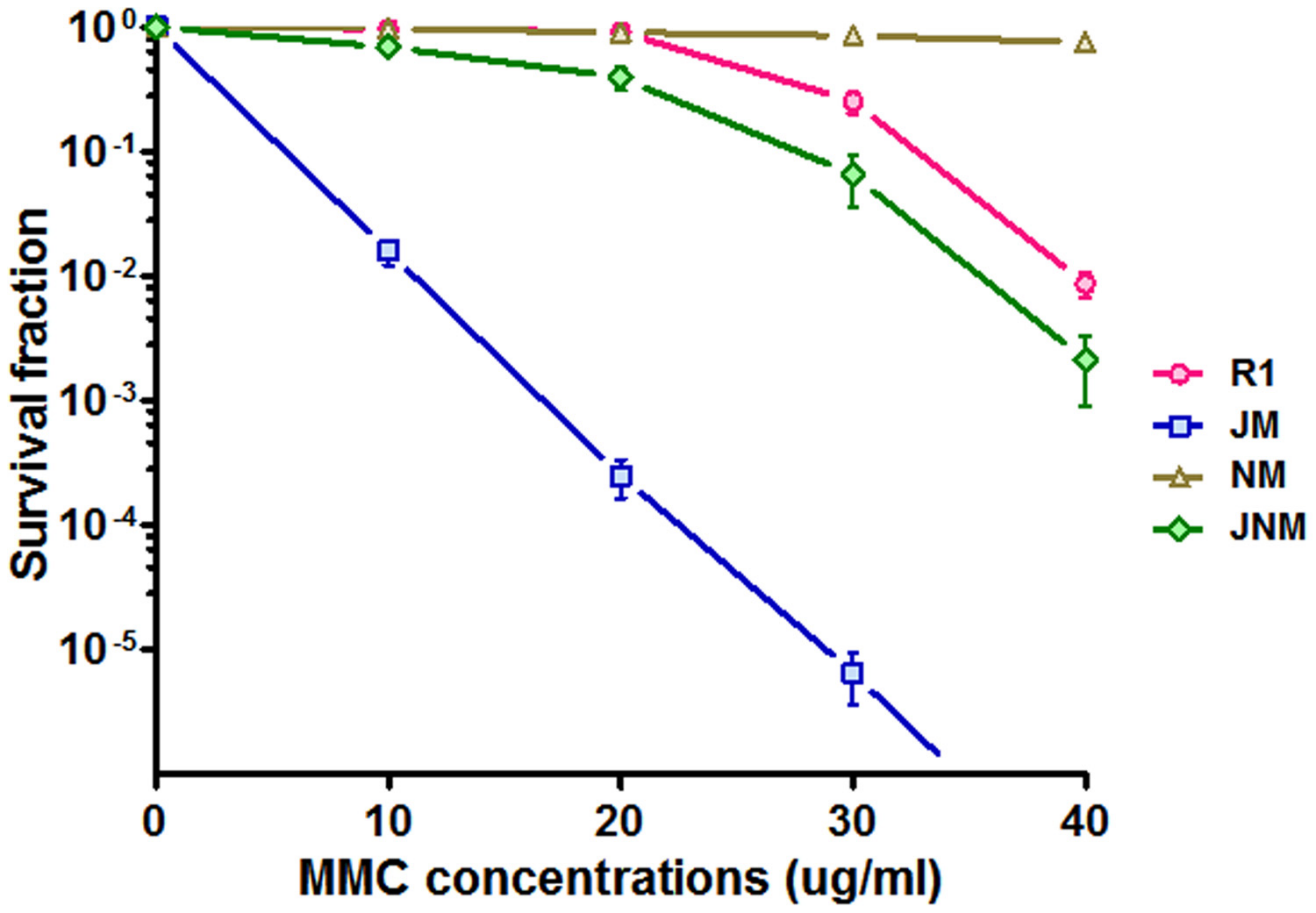
**Comparisons of phenotypes between *recJ* mutant and *nurA* mutant.** The survival fraction curves (treated with 10, 20, 30, and 40 ug ml^-1^ MMC) of different strains were plotted using GraphPad Prism 5 software. Data shown mean values from three independent experiments and bars depict the standard deviation (SD).

Because DrNurA could block DrRecJ nuclease activity, we were particularly interested in the functional relationships among these proteins. The phenotypes of *drrecJ* and *drnurA* mutants were compared. In contrast to dr*recJ* mutant, which showed growth defects and sensitivities to high temperature, and MMC treatment, *drnurA* mutants grew faster and were more resistant to MMC treatment (**Figure 6**; Supplemental Figure S6). The *drrecJ/drnurA* double mutant, on the other hand, displayed modest phenotype to high temperature and MMC treatment compared with the *drrecJ* mutant (**Figure 6**).

## Discussion

Despite that the RecJ/DHH superfamily proteins exist in bacteria, archaea, and eukaryotes, members of this family display various functions according to their different domain compositions. Typical RecJs, such as EcRecJ, exhibit 5′-3′ ssDNA exonuclease activity, possess DHH, DHHA1 and OB fold domains, and were suggested to join in numerous DNA repair processes (Handa et al., 2009a,b; Morimatsu and Kowalczykowski, 2014). Bacterial RecJ-like proteins, which only possess DHH and DHHA1 domains, have been confirmed to function as oligoribonucleases and 3′-phosphoadenosine 5′-phosphate (pAp) phosphatases, participating in nucleotide recycling (Wakamatsu et al., 2011). Archaea do not have typical RecJ proteins (Yuan et al., 2013), and the archaeal RecJ-like proteins have an intrinsic 3′-5′ ssRNA exonuclease activity and a 5′-3′ ssDNA exonuclease activity, which was recently identified to have proofreading function for 3′-mismatched RNA primers (Yuan et al., 2013). Prune, a cyclic AMPase in eukaryotes, could disrupt the phosphodiester bonds in cyclic nucleotides, possesses DHH and DHHA2 domains (Aravind and Koonin, 1998). Cdc45, one of the essential replication related proteins in eukaryotes, also processes DHH domains, but without phosphoesterase activity (Sanchez-Pulido and Ponting, 2011; Krastanova et al., 2012; Makarova et al., 2012). It is interesting to note the presence of uncharacterized CTDs in RecJs in the *Deinococcus-Thermus* phylum. The DrRecJ-core truncation mutant, corresponding to the RecJ-like protein, which only possesses DHH and DHHA1 domains, could not complement the *recJ* mutant, indicating that the *in vivo* roles of RecJ and RecJ-like proteins are different. Moreover, the DrRecJΔC truncation mutant, corresponding to the EcRecJ, which possesses DHH, DHHA1, and OB fold domains, but not the CTD, could not complement the *recJ* mutant as well, implying the CTD might play a critical role in the *Deinococcus-Thermus* phylum.

Homologous recombination generates error-free repair products and plays an important role in DSB repair and replication fork rescue processes (Longhese et al., 2010). The 3′ end resection is one critical process during HR, which is conducted cooperatively by nuclease and helicase (Longhese et al., 2010). In RecBCD deficient bacteria such as *D. radiodurans*, RecJ was suggested to be the key nuclease in 3′ end resection (White et al., 1999; Blasius et al., 2008; Cox et al., 2010). Despite that the classical EcRecJ lacking CTD function well *in vivo*, RecJs from *Deinococcus-Thermus* phylum exhibit uncharacterized CTDs. Our biochemical results showed that DrRecJ without CTD possess reduced nuclease activity, suggesting that the evolvement of this domain most likely contributes to *D. radiodurans* high HR efficiency. Bacteria in *Deinococcus-Thermus* phylum also possess NurA and HerA, which are identified as the essential nuclease and helicase in archaea 3′ end resection (Hopkins and Paull, 2008; Chae et al., 2012). However, our previous results suggested that *D. radiodurans* NurA and HerA contribute little to radiation resistance and have inhibitory effects on cell growth (Cheng et al., 2015). This implies that NurA and HerA might play other roles during HR or might even join in other pathways in these bacteria. The observation that DrHerA could stimulate DrRecJ nuclease activity through direct interaction with the DrRecJ-CTD suggests that DrHerA-NurA might has some functional connection with DrRecJ *in vivo*. It is interesting that HerA and NurA are present in almost all archaea but only in a few bacteria (Iyer et al., 2004). On the other hand, typical RecJ proteins are present in almost all bacteria but not in archaea. In *D. radiodurans*, the coexistence of HerA-NurA and RecJ, and the observed physical and biochemical relationship between DrRecJ-CTD and DrHerA suggest a novel regulation system.

Both RecJ and NurA could be stimulated by HerA and they block each other’s stimulation. While the co-binding of these three proteins indicates more than a competition relationship between RecJ and NurA. It is worth noticing that in addition to 5′-3′ ssDNA exonuclease activity, DrNurA also possesses 5′-3′ dsDNA exonuclease and endonuclease activity (Cheng et al., 2015). The opposite phenotypes between *recJ* and *nurA* mutants, further suggest these two proteins might play distinct roles *in vivo*. Further studies are required to determine if a modulation mechanism exists that HerA mediate the switching between RecJ and NurA performance *in vivo.* Moreover, SSB was reported to interact with NurA and block NurA nuclease activity in *Sulfolobus tokodaii* (Wei et al., 2008). SSB is another partner of DrRecJ, which could enhance DrRecJ nuclease activity (Jiao et al., 2012). Therefore, it is also worth seeing whether SSB also participates in this modulation process. Furthermore, HerA was reported to have interactions with Mre11, the bacterial sbcC ortholog, in *Sulfolobus acidocaldarius* and *S. tokodaii* (Hopkins and Paull, 2008; Quaiser et al., 2008; Zhang et al., 2008). Whether sbcCD has connection with HerA-NurA in *D. radiodurans* remained to be uncovered. Further investigation on other potential partners of DrRecJ, DrHerA and DrNurA and their interactions in *D. radiodurans* will provide a much more detailed modulation mechanism for cell proliferation and DNA repair processes.

## Materials and Methods

### Multiple Protein Alignment

There is a mistake for DrRecJ protein (coded by gene *dr_1126*) sequence annotation in NCBI website, and the correct one was given in the supplemental material (Supplemental Figure S1A). Sequences of TtRecJ, EcRecJ and DrRecJ-like (coded by gene *dr_0826*) proteins were obtained from NCBI website. Multiple alignments of full length RecJs and RecJ-CTDs were performed by Cobalt Constraint-based Multiple Protein Alignment Tool on NCBI website,^1^ followed by manual corrections. The CTD of DrRecJ was analyzed by HHpred online tool (Homology detection and structure prediction by HMM–HMM comparison^2^.

### Strains, Media and Transformation

All bacterial strains and plasmids used in this study are listed in Supplemental Table S1. All primers and Oligos used in this study are listed in Supplemental Table S2. *E. coli* or *D. radiodurans* strains were cultured and transformed as previously described (Cheng et al., 2015).

### Strains and Plasmids Construction

*drrecJ* and *drnurA* knockout strain were constructed in our previous work (Jiao et al., 2012). *drrecJ/drnurA* double knockout strain was constructed on the base of *recJ mutant.* Gene knockout was carried out using a deletion replacement method as described previously (Xu et al., 2008; Jiao et al., 2012). The DNA fragment for *nurA* deletion was amplified by upstream and downstream primers from *drnurA* knockout strain genomic DNA, followed by transformed into the *recJ* knockout strain. The double mutant strain (named as JNM) was screened with kanamycin containing TGY plates, and confirmed by PCR products analysis and sequencing. DNA fragments expressing the full length DrRecJ (705 aa), DrRecJΔC (region 1∼531 aa), DrRecJ-core (region 48∼431 aa) and full length EcRecJ (577 aa) were generated by PCR from *D. radiodurans* or *E. coli* (K-12) genomic DNA, using primers described in Supplemental Table S2. Fragments were cloned into the *Nde*I and *BamH*I sites of shuttle vector pRADK to construct complemented vectors. The *drrecJ* knockout was named as JM. The JM complemented with DrRecJ, DrRecJΔC, DrRecJ-core or EcRecJ were named as JM-J, JM-JΔC, JM-Jcore and JM-EcJ, respectively. Moreover, these fragments were also ligated into expression vector pET28b-HMT (Austin et al., 2009) at the *Nde*I and *Bam*HI sites to construct expression vectors. The constructed expression vectors contain HMT tag (6× His tag, maltose binding protein [MBP], and tobacco etch virus protease [TEV] cleavage site sequences) at N-terminal of these DNA fragments. Site mutations of *drrecJ* (D158A/H159A/H160A) and *drnurA* (D53A) were introduced by site-directed mutagenesis kit (Stratagene, USA), as described in reference (Jiao et al., 2012) and (Cheng et al., 2015). DrRecJ-like expression vector was constructed by ligating the *dr_0826* gene into the *Bam*HI and *Nde*I sites on pET28a expression vector. For pull down assays, *drherA* gene, *drherAΔN* fragment and *drnurA* gene were also ligated into pET28a expression vector.

### Western Blot Assays

*Deinococcus radiodurans* wild-type R1 or mutant strains were harvested when the cell density of the culture (OD_600_) reached 1.0. Cells were washed and lysed in PBS (added with 1 mg ml^-1^ lysozyme and 0.1% Triton-100) by sonication on ice. Proteins were separated on 12% sodium dodecyl sulfate polyacrylamide gel electrophoresis (SDS-PAGE) and transferred onto PVDF membranes (Millipore, USA). Rabbit anti-RecJ polyclonal antibody (prepared by our laboratory) was applied to measure the expression level of DrRecJ, DrRecJΔC, DrRecJ-core or EcRecJ of each strain, respectively. Rabbit anti-GroEL polyclonal antibodies (Sigma, USA) were used to measure the expression level of GroEL as controls. HRP-conjugated goat anti-rabbit antibody (Beyotime Biotechnology, China) was used as secondary antibody and signal was detected by SuperSignal West Pico Chemiluminescent Substrate (Thermo scientific).

### Co-immunoprecipitation

One liter of the wild type R1 culture was harvested when cell density (OD_600_) reached 2.0. Cells were suspended with PBS (added with 1 mg ml^-1^ lysozyme and 0.1% Triton-100), followed by incubation on ice and sonication. After centrifugation at 15,000 *g* for 40 min, the supernatant was incubated with anti-RecJ antibody and Protein G Sepharose beads at 4°C overnight. The beads are then washed three times with PBS containing 0.1% Triton-100 to remove non-specific binding proteins. And the antibody, bait, and target proteins are eluted by boiling and analyzed by 12% SDS-PAGE.

### Protein Expression and Purification

Wild-type and mutated DrRecJ were expressed and purified as previously described (Jiao et al., 2012). DrRecJ, DrRecJΔC, DrRecJcore and EcRecJ with HMT tags were expressed in *E. coli* BL21 (DE3) pLysS cells (Transgen biotech, China) at 30°C for 5 h with induction of 0.5 mM IPTG when the OD_600_ reach 0.8. The cells were re-suspended in lysis buffer A (20 mM Tris-HCl [pH 8.0], 500 mM NaCl, 10% (v/v) glycerol and 1 mM β-mercaptoethanol) containing protease inhibitor cocktail (Roche Biochemicals, Switzerland) and lysed by sonication. After centrifugation at 15,000 *g* for 40 min, the supernatant was loaded onto a Ni-NTA column (GE Healthcare, USA), which was pre-equilibrated with lysis buffer. Target protein was eluted with elution buffer B (20 mM Tris–HCl [pH 8.0], and 500 mM NaCl, and 300 mM imidazole). The collected fraction was digested by TEV protease at 4°C overnight. Amylose column was used to remove the HMT tag. Then the protein was dialyzed against buffer C (20 mM Tris-HCl, pH 8.0, 100 mM NaCl, 10% (v/v) glycerol, 1 mM EDTA and 1 mM DL-Dithiothreitol [DTT]), and loaded onto Hitrap Q ion exchange column (GE Healthcare). Target protein was eluted by gradient elution. Among them, DrRecJ, DrRecJΔC and EcRecJ was dialyzed against buffer C again and purified by HiTrap Heparin HP column (GE Healthcare). Finally, proteins were further purified by Superdex 200 (or 75) column (GE Healthcare) with buffer C. Fractions containing the target proteins were pooled, concentrated, and flash frozen in liquid nitrogen, and stored at -80°C.

Wild-type or HAS-domain deleted DrHerA, and DrNurA proteins were expressed and purified according to the reference (Cheng et al., 2015). The DrHerA, DrHerAΔN, DrNurA and RecJ-like proteins with N-terminal 6× his tag were purified by Ni-NTA (GE) affinity column, Hitrap Q (GE) ion exchange column and Superdex 200 (or 75) (GE) chromatography, and stored in buffer C at -80°C. The purity of each protein was checked by silver stained SDS-PAGE.

### Immunodot Blotting Assay

Immunodot blotting assay was performed as previously described (Cheng et al., 2014), with some modifications. Ten nanomolar DrRecJ, DrRecJΔC, DrRecJ-core, EcRecJ, DrHerA or DrNurA were spotted on a nitrocellulose (NC) membrane. Lysozyme was also spotted as negative control. The membranes were blocked in TBST containing 5% non-fat milk powder at 4°C for 2 h, followed by incubation in 1 μM purified DrRecJ, DrRecJΔC, DrRecJ-core, EcRecJ or DrHerA protein (100 mM NaCl, 20 mM Tris-HCl, [pH 7.5], 1 mM DTT, and 1 mM EDTA) at 4°C overnight. Membranes were washed by TBST (TBS containing 0.05% Tween 20) for three times, followed by incubation with the primary antibody, anti-RecJ or anti-HerA (prepared in our laboratory) with 1:1000 dilution, at 4°C for 4 h. Again, membranes were washed by TBST for three times and subsequently incubated with the secondary antibody HRP-conjugated goat anti-rabbit (Beyotime Biotechnology, China) with 1:10,000 dilution at 4°C for 4 h. Finally, the membranes were washed another three times with TBST and signals were detected by SuperSignal West Pico Chemiluminescent Substrate (Thermo scientific).

### Pull Down Assay

Pull down assays were performed as previously described with some modifications (Cheng et al., 2015). Two hundred micro liter bait protein (0.5 mM) was incubated with 20 μl Ni-NTA agarose beads (GE) and washed three times by washing buffer (100 mM NaCl, 20 mM Tris-HCl [pH 7.5], 0.05% Tween 20) and then incubated with 400 μl 0.5 mM DrNurA, DrRecJ, DrRecJΔC, DrHerA, or DrHerA/DrRecJ (with lysozyme as control) at 4°C for 3 h. The beads were washed by washing buffer a few times as far as the lysozyme is completely washed off. Proteins were eluted by 50 μl elution buffer (500 mM imidazole, 100 mM NaCl, 20 mM Tris-HCl [pH 7.5]) and analyzed by 12% SDS-PAGE.

### Nuclease Activity Assays

Oligo 3 (GTCCAGGCTCTCGTTCAGGGTCTTTTTGGTG), and 5′ FAM fluorescence labeled Oligos (O1: AAAAAAAAAA; O2: TGATGAAAGCCAATCCACCAAAAAGACCCTGAACGAGAGCCTGGAC) were synthesized by Sangon Biotec (China) and purified with PAGE. 5′ overhang DNA substrate was obtained by annealing O2 and O3. For RecJ nuclease activity, when DrHerA was added, DrRecJ (5 nM, monomer) was preincubated with various concentrations of DrHerA (5, 20, and 80 nM, hexamer), followed by adding 100 nM DNA substrate in reaction buffer (25 mM Tris-HCl [pH 7.5], 60 mM KCl, 1 mM DTT, 0.1 mg ml^-1^ BSA). To initiate the reaction, 0.1 mM MnCl_2_ was added. The mixtures were incubated at 37°C for 20 min (for time course experiments, different incubation time points were used). For NurA nuclease activity, reaction mixtures containing DrNurA-HerA complex (200 nM, complex) with or without various concentrations of DrRecJ (D158A/H159A/H160A) and 100 nM substrate in reaction buffer (25 mM Tris-HCl [pH 7.5], 60 mM KCl, 1 mM DTT, 5 mM MgCl_2_, 10 mM MnCl_2_, 0.1 mg ml^-1^ BSA) were incubated at 37°C for 30 min. Reactions were stopped by adding the same volume of 2× reaction stop buffer (95% formamide, 50 mM EDTA, 0.05% SDS, 0.01% Bromophenol blue), followed by boiling at 100°C for 5 min and flash cooled on ice for 10 min. Reaction products were analyzed on 15% denaturing polyacrylamide gels containing 7 M urea in TBE buffer. Gels were imaged by fluorescence mode (FAM) on Typhoon FLA 9500 (GE) and bands were analyzed by Image J Software (National Institutes of Health, USA), if necessary.

The kinetic parameters of DrRecJ activity were determined according to the method of reference (Sharma and Rao, 2009; Zhao et al., 2013). Five nanomolar RecJ (in the presence or absence of 500 nM DrHerA) was incubated with increasing concentrations of O2 substrate (20, 30, 40, 50, 60, 75, 100, 200, 300, 400, and 500 nM) in reaction buffer for 30 min at 37°C. The reaction was stopped by 2× reaction stop buffer and boiling at 100°C for 5 min followed by flash cooling on ice for 10 min. The reaction mixture was electrophoresed, and the gels were imaged by FAM on Typhoon FLA 9500 (GE) and bands were analyzed by Image J Software (National Institutes of Health, USA). The velocity of the reaction was calculated from the concentration of undegraded substrate, which was then plotted against the total substrate concentration to determine the *K_m_* and *V_max_* values using the Lineweaver–Burk equation. The enzyme activity data were plotted in GraphPad Prism 5 as the mean of at least triplicate determinations, with error bars representing standard deviation.

### Electrophoresis Mobility Shift Assay

Electrophoresis mobility shift assays (EMSA) were performed as previously described (Cheng et al., 2014). Twenty micro liter reaction mixtures containing 100 nM 10 nt ssDNA was incubated with various concentrations of DrRecJ (0, 0.2, 0.4, 0.8, and 1.6 μM) in binding buffer (80 mM NaCl, 20 mM Tris-HCl, [pH 7.5], 1 mM DTT) at 30°C for 10 min. To analysis the influence of different proteins on DrRecJ ssDNA binding ability, 1 μM DrHerA, DrHerAΔN or DrNurA were added in the system. Samples were separated by electrophoresis on 5% TBE native-PAGE. Gels were imaged by FAM on Typhoon FLA 9500 (GE) and bands were analyzed by Image J Software (National Institutes of Health, USA), if necessary.

### Growth Curve Assays

Growth curve and temperature sensitive assays were performed as previously described (Jiao et al., 2012). Briefly, after the cell density of the culture (OD_600_) reached 1.0, 1 ml aliquots were re-suspended in 100 ml new fresh TGY medium and incubated at 30°C, or 37°C. The cell growth rate was monitored by measuring OD_600_ at various incubation times. Three independent experiments were performed for each strain. Growth curves were plotted by GraphPad Prism 5.0.

### DNA Damage Agents’ Survival Rate Assays

Growth curve and temperature sensitive assays were performed as previously described Cells were grown in TGY media to early exponential phase (OD_600_ = 0.6∼0.8). For mitomycin C (MMC) treatment, cells were incubated with various concentrations (10, 20, 30, and 40 mg ml^-1^) of MMC at 30°C for 20 min, and then diluted to appropriate concentrations and plated on TGY plates. Three independent experiments were performed for each strain. Colonies were counted after cultured at 30°C after 3 days. Survival rate curves were plotted by GraphPad Prism 5.0.

## Conflict of Interest Statement

The authors declare that the research was conducted in the absence of any commercial or financial relationships that could be construed as a potential conflict of interest.

## Footnotes

1 http://www.ncbi.nlm.nih.gov/tools/cobalt

2 http://toolkit.tuebingen.mpg.de/hhpred
