# Supplementary material for: A Novel C-Terminal Domain of RecJ is Critical for Interaction with HerA in Deinococcus radiodurans
Source: Front Microbiol. 2015 Nov 30;6:1302. doi: 10.3389/fmicb.2015.01302 (PMC4663267; doi:10.3389/fmicb.2015.01302)
Supplement: Supplementary file 1 [file Data_Sheet_1.PDF]

Supplemental tables and Figures

Table s1 Strains and plasmids used in this experiment

Strain and plasmid	Relevant feature	Reference or source
**Strains**		
E.coli		
**DH(5α)**	*supE44*, *ΔlacU169 (φ80lacZΔM15)*, *hsdR17, recA1, endA1, gyrA96, thi-1, relA1*	Invitrogen
**BL21(DE3) plysS**	F^-, *ompT*, $hsdS_B$ $(r_B^- m_B^-)$, *dcm (DE3), gal (λcI857,ind1,Sam7,nin5,lacUV5-T7gene1)*	Invitrogen
D.radiodurans		
**R1**	Wild-type strain	ATCC13939
**JM**	R1 but *recJ::str*	(1)
**JM-J**	JM but compensated with pRAD-*recJ*	(1)
**JM-J△C**	JM but compensated with pRAD-*recJ△C*	This study
**JM-Jcore**	JM but compensated with pRAD-*recJ-core*	This study
**JM-EcJ**	JM but compensated with pRAD-ec*recJ*	This study
**NM**	R1 but *nurA::kan*	(2)
**JNM**	R1 but *nurA::kan, and recJ::str*	This study
**Plasmids**		
**pMD18-T vector**	For TA cloning, *lacZ*, Amp^r	TaKaRa
**pET28-HMT**	T7 promoter, T7 transcription start, His•Tag coding sequence, T7•Tag coding sequence, Multiple cloning sites(BamH I - Xho I), T7 terminator, *lacI* coding sequence, pBR322 origin, Kan^r, f1 origin, 6His-tag coding sequence, Maltose Binding Protein, TEV protease site	(1)
**HMTN**	As pET28-HMT but ligated with *nurA*	(2)
**HMTH**	As pET28-HMT but ligated with *herA*	(2)
**HMTHC**	As pET28-HMT but ligated with *herA△N*	(2)
**pET28aN**	pET28a ligated with *nurA*	(2)
**pET28aH**	pET28a ligated with *herA*	This study
**pET28aHC**	pET28a ligated with *herA△N*	This study

**pRADK**	*E. coli–D. radiodurans* shuttle vector	Laboratory stock
**pRAD-*recJ***	As pRADK but *kan*^{r} was replaced with *drrecJ* gene	(1)
**pRAD-*recJ*⊿*C***	As pRADK but *kan*^{r} was replaced with *drrecJ* ⊿*C* fragment	This study
**pRAD-*recJcore***	As pRADK but *kan*^{r} was replaced with *drrecJ-core* fragment	This study
**pRAD-ec*recJ***	As pRADK but *kan*^{r} was replaced with *ecrecJ gene*	This study

Table s2 Primers or oligonucleotides used in this study

Oligonucleotide name	Sequence (5'→3') *
**nurA-F(NdeI)**	TTTTTTCATATGCGTATTCGTCTGGAC
**nurA-R(BamHI)**	TTTGGATCCTCACGCCACCACCCC
**herA-F(NdeI)**	TTTTTTCATATGACGGGGAATGACGTGCAAG
**herA-R(BamHI)**	TTTGGATCCTCAGCGCAGCAGCCCCG
**herA(N-135)-F(NdeI)**	TTTTTTCATATGGCCGCGTTTCCCGGTG
**nurA D53A-F**	TGCTGGTGGTGGCCGGCAAACCCCG
**nurA D53A-R**	CGGGGTTTGCCGGCCACCACCAGCA
**recJ-F(NdeI)**	TTTTTTCATATGAGCCGGCCTGCCCACTGG
**recJN48-F(NdeI)**	TTTTTTCATATGCTAGAACTGACGCCCAACCCGG
**recJ-R(BamHI)**	TTTGGATCCTTAACCCGCAGCGAGCGCCAG
**recJN431-R(BamHI)**	TTTGGATCCTCAAGCCGGGACCGGGGTCGG
**recJN531-R(BamHI)**	TTTGGATCCTCAGCGCAGCGCCGCCGCGT
**RecJ D158A/H159A/H160A-F**	GTTCTCGCCCGGCGCGGCGGCGGCGGTGACCACGACCTC
**RecJ D158A/H159A/H160A-R**	GAGGTCGTGGTCACCGCCGCCGCCGCGCCGGGCGAGAAC
**ecrecJ-F(NdeI)**	TTTTAACATATGAAACAACAGATACAACTTCG
**ecrecJ-R(BamHI)**	TTTGGATCCCTAAATTGGCCAGATATTGTC
**dr0826-F(NdeI)**	TTTTTTCATATGACCGCCGAAAACTCTGCCAC
**dr0826-R(BamHI)**	TTTGGATCCTTACTCTGCCGTCGCCTGCGC
**O1 (5'FAM)**	AAAAAAAAAA

**O2 (5'FAM)**	TGATGAAAGCCAATCCACCAAAAAGACCCTGAACGAGAGCCTGGAC
**O3**	GTCCAGGCTCTCGTTCAGGGTCTTTTTGGTG

*Underlining indicates restriction sites

Reference

1. **Austin BP, Nallamsetty S, Waugh DS.** 2009. Hexahistidine-tagged maltose-binding protein as a fusion partner for the production of soluble recombinant proteins in Escherichia coli. Methods in molecular biology **498:**157-172.
2. **Cheng K, Chen X, Xu G, Wang L, Xu H, Yang S, Zhao Y, Hua Y.** 2015. Biochemical and Functional Characterization of the NurA-HerA Complex from Deinococcus radiodurans. Journal of bacteriology **197:**2048-2061.

Supplemental Figures

Figure 1s

A

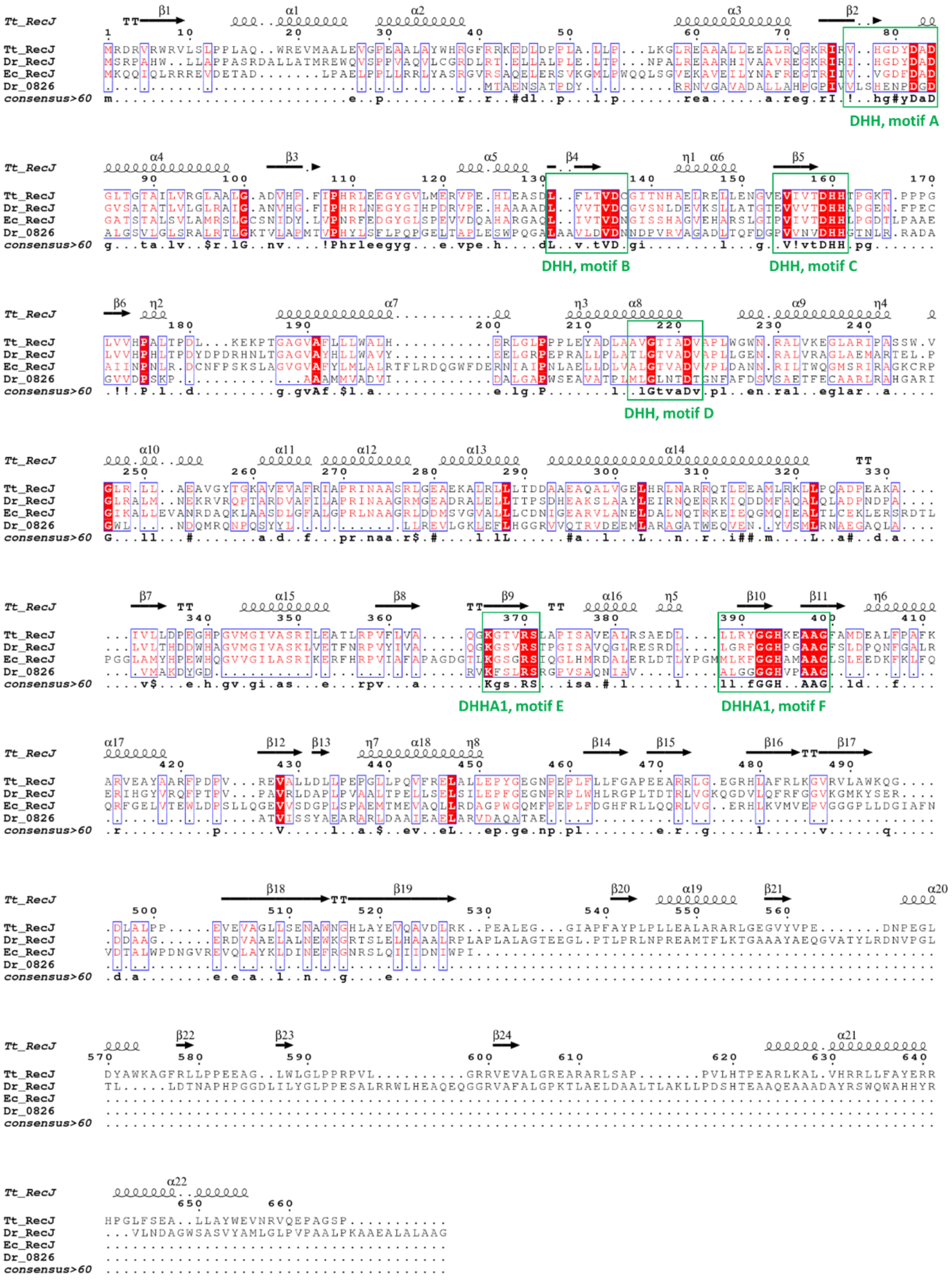

B

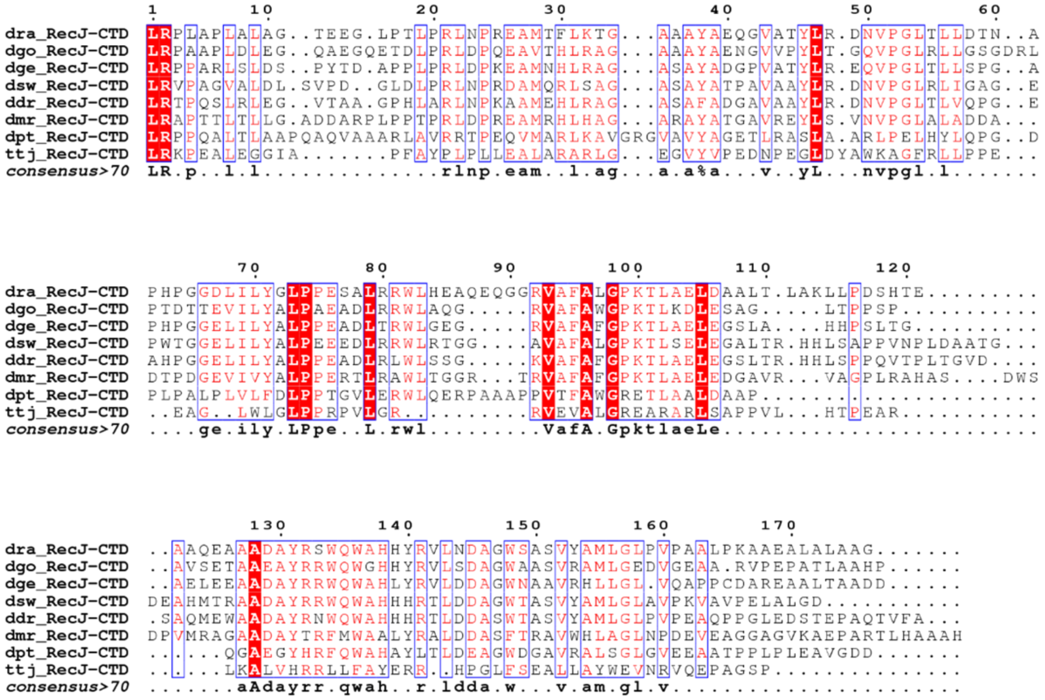

C

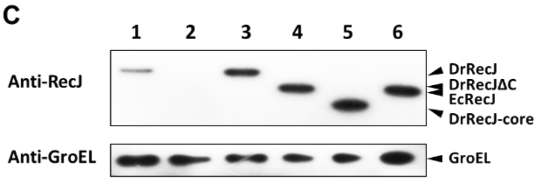

Figure s1. Domain analysis and complementation verification.

A, Alignment of some representive RecJ/DHH protein superfamily members. DR, *D. radiodurans;* TT, *Thermus thermophiles;* EC, *E. coli*. DR_0826 stands for the RecJ-like protein in *D. radiodurans*. Conserved motifs in DHH domain and DHHA1 domain were marked with frames.

B, Alignment of RecJ-CTDs. dra, *D. radiodurans;* dgo, *Deinococcus gobiensis*; dge, *Deinococcus geothermalis*; dsw, *Deinococcus swuensis*; ddr, *Deinococcus deserti*; dmr, *Deinococcus maricopensis*; dpt, *Deinococcus proteolyticus*; ttj, *Thermus thermophilus HB8*.

C, Western blot analysis of different strains' RecJ expression levels.

Lane 1~6: The signals of RecJ (or GroEL) in wild type strain R1, *drrecJ* knockout strain, DrRecJ complemented strain, DrRecJΔC complemented strain, DrRecJ-core complemented strain and EcRecJ complemented strain lysis.

Figure s2

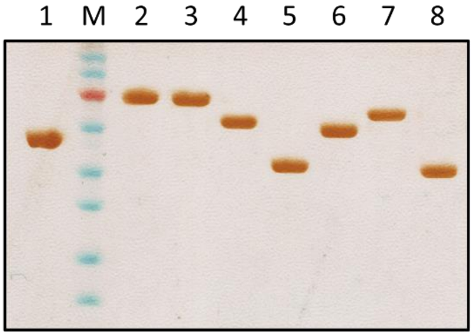

Figure s2. Protein purity checked by silver stained SDS-PAGE.

M: Marker (150, 100, 70, 50, 35, 25, 20 and 15 kDa); Lane 1~8: DrHerAΔN, DrRecJ, DrRecJ (D158A/H159A/H160A), DrRecJΔC, DrRecJ-core, EcRecJ, DrHerA, DrNurA.

Figure s3

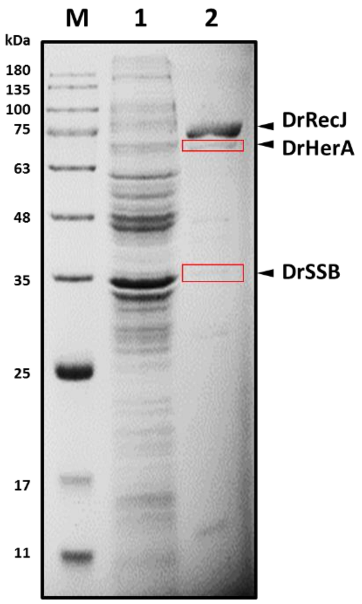

Figure s3. SDS-PAGE analysis of proteins co-immunoprecipitated by DrRecJ.

Lane 1: The total protein of *D. radiodurans*; Lane 2: The proteins immunoprecipitated by anti-RecJ antibody. The positions on gel marked by arrows were analyzed by mass spectrometry and identified as DrRecJ, DrHerA and DrSSB, respectively.

Figure s4

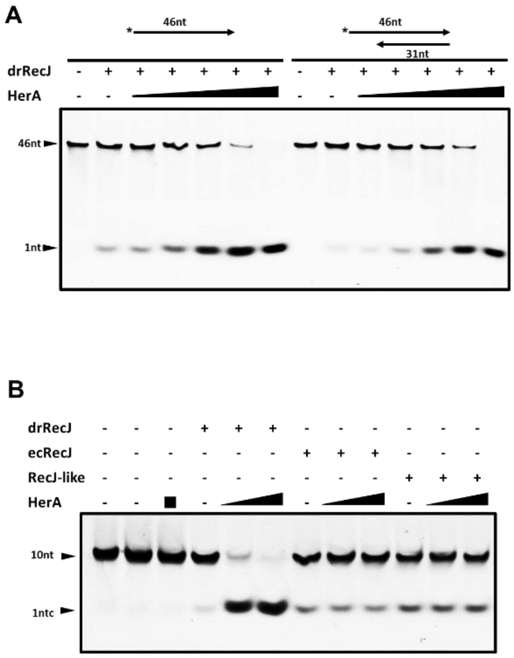

Figure s4. DrHerA stimulation is non-substrate-specific but protein-specific and species-specific.

A, DrHerA has stimulation for DrRecJ on much longer ssDNA substrate and 5’ overhanging DNA substrate.

B, DrHerA has no stimulation on EcRecJ and *D. radiodurans* RecJ-like protein. 10 nt ssDNA was used as substrate for digestion. The nuclease activities of EcRecJ and DrRecJ-like protein on 10 nt DNA were compared in the absence or presence of DrHerA, using same reaction conditions as DrRecJ, except that 10 mM Mg^{2+} was used for EcRecJ activity and 5 mM Mn^{2+} was used for RecJ-like activity.

Figure s5

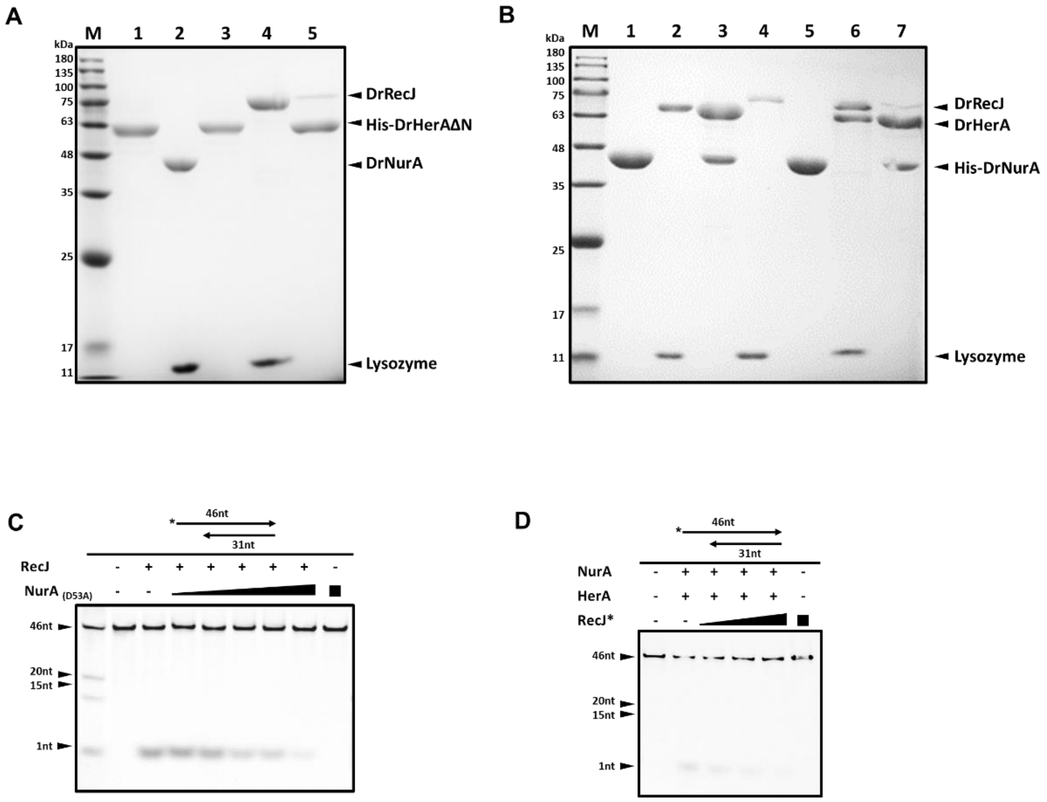

Figure s5. Pull down assays and nuclease assays to study the relationships among DrRecJ, DrHerA and DrNurA.

A, DrHerA ΔN still display interaction with DrRecJ while no longer interact with DrNurA. 400 μl 0.5 mM DrNurA, or DrRecJ (with lysozyme as control) was incubated with His-DrHerA ΔN bound Ni-NTA beads and analyzed by 12% SDS-PAGE. Lane 1: His-DrHerA ΔN; Lane 2: DrNurA + lysozyme (input control); Lane 3: DrNurA pulled by His-HerA; Lane 4: DrRecJ + lysozyme (input control); Lane 5: DrRecJ pulled by His-DrHerA ΔN.

B, Pull down assays showed that co-binding exists among DrRecJ, DrHerA and DrNurA. 400 μl 0.5 mM DrHerA, or DrRecJ (with lysozyme as control) was incubated with His-DrNurA bound Ni-NTA beads and analyzed by 12% SDS-PAGE. Lane 1: His-DrNurA; Lane 2: DrHerA + lysozyme (input control); Lane 3: DrHerA pulled by His-DrNurA; Lane 4: DrRecJ + lysozyme (input control); Lane 5: DrRecJ pulled by His-DrNurA; Lane 6: DrHerA+DrRecJ + lysozyme (input control); Lane 7: DrHerA and DrRecJ pulled by His-DrNurA.

C, High concentration of DrNurA could inhibit DrRecJ nuclease activity. 100 nM DNA substrate and 10 nM RecJ was used. DrRecJ DNA nuclease activity was analyzed in the absence or presence of DrRecJ in various molar ratios (RecJ monomer: NurA dimer= 1:10, 1:40, 1:80, 1:160 or 1:320).

D, DrRecJ inhibit DrHerA stimulation on DrNurA nuclease activity. 100 nM DNA substrate and

500 nM DrNurA-HerA complex was used. DrNurA-HerA DNA nuclease activity was analyzed in the absence or presence of DrRecJ in various molar ratios (NurA-HerA complex: RecJ monomer = 1:1, 1:2 or 1:4). RecJ* represented the inactive DrRecJ protein RecJ (D158A/H159A/H160A).

Figure s6

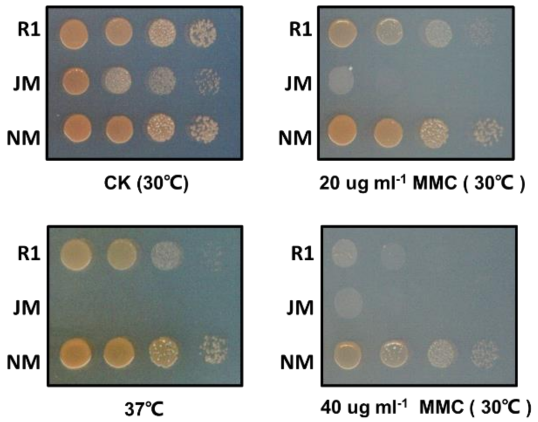

Figure s6. Comparisons of phenotypes between *recJ* mutant and *nurA* mutant.

Growth and MMC resistance features of wild type R1, *drrecJ* mutant and *drnurA* mutant strains. The diluted bacteria suspensions (treated with 20 ug ml^{-1} or 40 ug ml^{-1} MMC or not) were spotted on TGY medium and cultured at 30◦ C and 37◦ C.
